# The Anatomy of the Arteries of the Human Body; with Its Applications to Pathology and Operative Surgery; in Lithographic Drawings: With Practical Commentaries

**Published:** 1841-01

**Authors:** 


ART. XIV.

*The Anatomy of the Arteries of the Human Body; with its Applications to Pathology and Operative Surgery; in Lithographic Drawings: with Practical Commentaries.* By RICHARD QUAIN, Professor of Anatomy in the University College. *The Delineations* by JOSEPH MACLISE, Esq., Surgeon.—*London,* 1840. Parts I-II. 8vo, pp. 90. *Super-royal Folio, Ten Plates.*

THIS is a work excellent both in its conception and execution, and meriting the warmest patronage of the profession. Its plan and object cannot be better stated than in the author's own words:

"Several years have elapsed since I became impressed with the belief that the difficulties which have often occurred in the performance of those surgical operations in which the larger arteries are concerned, have arisen in great part from want of sufficient acquaintance with the differences in anatomical disposition to which these vessels are subject—not merely those deviations in the origin of large branches, which are usually named varieties, but other peculiarities of various kinds which are liable to occur, such as those which affect the length, position, or direction of the vessels. Under that impression I was led to observe these circumstances more closely, and finally determined to obtain a record of the condition, whatever it might be, of the more important vessels in a considerable number of cases—a record to be made especially with a view to points bearing on practical surgery.

"With this view, I examined with more or less attention the bodies which were received during a series of years for the study of anatomy into the School of Medicine in University College. These bodies, to the number of 930, were with rare exceptions so inspected with reference to the subject of my enquiries, that anything very unusual could not escape notice; and, in order to insure accuracy, when other occupations allowed, the arteries were carefully examined and their condition noted at the time, attention being always particularly directed to those vessels and to the points in their history which seemed to be of importance in the practice of surgery. This detailed investigation was continued until the number of cases observed appeared such as would afford grounds for reasonable conclusions both as to the limits of the deviations from the ordinary standard, and as to the relative frequency of their occurrence. At the same time that the observations thus made were written down, drawings were obtained of all the important peculiarities which presented themselves, and when it was practicable the preparations were preserved. The varieties in the arrangement of the blood-vessels thus noted grew, as may be supposed, to be very numerous; but instead of difficulties multiplying with the number of observations, it was usually found that as the facts accumulated, the transition from one state to a very different one ceased to be abrupt or without method, for others from time to time interposed which served to link them together.

"Originally these observations were intended exclusively for the benefit of my class; but as their number and connexion seemed likely to render them more extensively useful, I resolved to publish them. On examining with a view to publication the materials which I had collected, it became obvious that their utility would be very limited, unless as a part of a full history of the arteries with adequate delineations. In consequence, a series of drawings, showing the arteries according to their usual arrangement, has been prepared, and to these are appended the observations previously alluded to. The work has thus grown under my hands, and has gradually assumed its present form. To carry out my views as to the delineations, I obtained the assistance of my friend and former pupil, Mr. Joseph Maclise. In reference to that gentleman's labours, it may be allowed me to say, that while I have had the co-operation of an anatomist and surgeon, obviously a great advantage, the drawings will, I believe, be found not to have lost in spirit or effect.....

"In the present work, the arteries are in the first place represented according to their most frequent arrangement, without the accompanying veins and nerves. 2dly. They are shown in connexion with the larger veins and nerves. 3dly. The deviations from that which has been taken as the standard, because the most frequent condition of the arteries are illustrated in a series of sketches. Into this part of the subject as much of arrangement or classification has been introduced as my information admits. 4thly. Such peculiarities in the veins, and occasionally of the nerves and muscles, as appeared likely to be of importance in surgical operations are represented on a reduced scale. 5thly. At the end of the publication will be given illustrations of the state of the arteries after the operations for aneurism.

"The letter-press, besides an explanation of the drawings and remarks on them, will contain: 1. A series of Tables showing, in a considerable number of cases, the condition of the arteries as to some of the points of most importance in their anatomy. 2. Practical Commentaries; which will consist for the most part of inferences from the facts previously set forth, and their application in performing surgical operations." (Preface, pp. 5, 6, 8.)

The only previous works on the same subject with which Professor Quain's publication can be compared, are Haller's *Icones Anatomicæ*, Scarpa's plates in the treatise *Sull' Aneurisma*, and Tiedemann's *Tabulæ Arteriarum;* and it is no small praise to say that it can well bear the comparison; indeed, it is but justice to state that while it is as original and as accurate as the best of these works, it is far superior to them all in its relation to practical surgery. Judging from the specimens before us, we believe that it will not only give us a much more accurate and more complete anatomy of the whole arterial system than we already possessed, but that it will give all the most precise information relating to every point connected with the blood-vessels and nerves, which it most imports the practical surgeon to have. The plates do the highest credit to Mr. Maclise as works of art. In addition to their beauty and accuracy, they have the great advantage of representing the objects of their natural magnitude, a point of first-rate importance in surgical anatomy. Most of them are also coloured.

Considering these circumstances, it is surprising that the work can be offered to purchasers at so low a rate as twelve shillings per part, (each containing five plates;) but we presume, the author, like his distinguished predecessors in the same path, had higher objects in publishing it than mere emolument. We, however, consider it our duty most strongly to recommend the work to our readers for its intrinsic merits, and with a view to their own advantage; and we shall feel more regret for the loss of credit to the profession, than for any pecuniary loss that may be sustained by the author, should it fail to obtain that ample encouragement and wide circulation which it so richly merits. At any rate, should the work proceed as it has commenced, and of this we have no doubt, it will constitute an enduring memorial of the merits of its authors, ranging in the same class with the productions of William Hunter and Joseph Swan, of which England has such just reason to be proud.

